# General Remarks on Aliments Produced by the Different Classes of the Animal Kingdom, and Their Influence on the Human Body

**Published:** 1800-01

**Authors:** J. J. Virey

**Affiliations:** Val de Grace


### *General Remarks on Aliments produced by the different Claffes of the Animal Kingdom, and their Influence on the Human Body. By* J. J. Virey, *of the Val de Grace.*

[Read to the Medical Society of Paris, the 7th Meffidor, 6th Year.]

Non aer, non pontus, non tellus, denique cuncta

Sufficiunt noftræ vix elementa gulae.

THE nature of the aliment with which we are furnifhed by every fpecies of animals, has not hitherto been inveftigated with that attention which the importance of the fubject requires; either the practical application of chemical knowledge has been neglected, or the opinion of the ancients, that crude refult of an obfcure and verbofe fyftem of phyfic, has been confounded with facts. Supported by found experience, we fhall attempt to travel over this thorny path with rapidity.

Sed quanto plura reftant, quantoque mirabiliora inventu! Illa enim majore parte cibi, aut odoris decorifve commendatis ad numerofa experimenta duxit.

It is erroneous to maintain, that the nutritive quality is principally in the gelatinous fubftance of animals, fince this is much lefs nourifhing than the albuminous fubftance. The latter, when entirely animalifed, requires only a light exercife, and a greater degree of oxygenation, to be converted into fibrous fubftance; but the gelatinous matter, being analogous to the mucilaginous fubftance of vegetables, requires a greater effort of nature for affimilation, and a more confiderable feparation of hydrogen and carbon. Befides, herbivorous animals cannot endure hunger for any confiderable length of time, while the carnivorous can fuftain it with lefs difficulty for feveral days; for inftance, dogs, the different fpecies of cats, birds of prey, and many of the farcophagous infects.

Though there are fome nations who fubfift entirely on vegetables, and man being naturally more granivorous than carnivorous, as, according to Brouffonet, his teeth have the proportion =20 : 12; it would neverthelefs be impoffible for him to abandon his animal regimen in the northern climates of the globe, without reducing himfelf almoft to diffolution. It is well known that animal food imparts much vigour to man, and that the ichthyophagous nations, fuch as the Chinefe, the Dutch, and others, are very prolific.

The animal fubftances which yield moft aliment, appear to be thofe which in their ftructure approach neareft to that of the human form. The lefs perfect the animal is, the lefs it yields nutrition to man. This is manifefted by examining the feries of thefe creatures, and comparing their different fubftances. The albumen abounds only in thofe animals which have red blood, and particularly in thofe whofe blood is warm. In defcending the fcale of animalifation, the albumen gradually difappears. At firft, it is fucceeded by a ftrong jelly, which becomes more and more attenuated in other animals; and that which forms the whole body of the zoophytes, may be faid to be an infpiffated ferum.

The mammiferous animals, and birds with red and warm blood, approach neareft to that of man, and have a confiderably larger quantity of albumen, a more fubftantial jelly, and their mufcular fibres abound more with azote and carbon than any of the other claffes. The reafon of this is, that their blood is of a more fibrous nature, and its proportionate mafs is ftronger. Their fat contains more of the mild principle difcovered by Scheele. But the mufcles of all carnivorous animals are too hard, in confequence of their manner of living, and becaufe their humours are too much inclined to putrefaction, to furnifh an agreeable and nourifhing food. The fkin, inteftines, and eggs of birds, and aquatic mammiferous animals, have a fmell like that of mire, and a difgufting putrefcency. They have much blood, and their fat is oily and abundant. The other animals of thefe two claffes, particularly the nibbling and ruminating kinds, as well as the gallinaceous, and fparrows, (birds which Linnæus compares with the laft-mentioned) furnifh the moft agreeable and palatable food. The yolk of the eggs eggs of aquatic birds, is proportionably lefs valuable and delicate than that of other fowls. The milk of animals that chew the cud, is the only kind from which good cheefe can be made. It is probable that the digeftive organs of this order, which give to their fat the febaceous confiftence, likewife increafe the cafeous and butyraceous matter in their milk. I have obferved that the milk of bitches was rather thick, and had the difagreeable tafte common to that of all carnivorous animals.

All flefh of the fame clafs of animals, however, is not equally nutritive. The flefh of young animals affords lefs nourifhment than that of old ones; and this difference is much more obvious in hot than in cold climates. There is alfo another difference, which confifts in the greater or lefs degree of vigour the aliment of animals imparts to thofe who ufe it. This arifes from the various degrees of ftimulation which animal food exerts in the human body. It is remarkable, that certain kinds of flefh, without the aid of fpices, or feafoning, increafe the tone and vigour of the fyftem. ATHENÆUS mentions an athletic Theban, who by living on goat's flefh, became more robuft than others who fubfifted on pork, which is flabby and too fat. The goat-herds, who eat much animal food, fometimes even in a raw ftate, are of an athletic conftitution. The ancient Germans, the Bretons, the Tartars or Scythians, the Efquimaux, and even the people of the fouthern climates, as the Ethiopians and Arabs, confirm this obfervation. Alexander Selkirk, a native of Normandy, who had been much reduced, acquired an incredible degree of ftrength by living on raw flefh in the Ifle of Juan Fernandez; but relapfed into his former debility, by adopting the regimen of polifhed nations. The fame effect is obfervable in other carnivorous people. It feems as if a portion of the life of the animal that expires under the teeth of the devourer, paffes into his ftomach. This diet is particularly neceffary to the Northern nations, who require the moft powerful ftimulants, and part of whofe lives is, as it were, confumed by continual cold. We alfo find feveral perfons, particularly among the Tartar Kalmuks, who have ftronger, fharper, and more pointed teeth, and the *molares*, or grinders, fometimes lefs numerous than among the negroes.* The prominent teeth of the latter appear to me to indicate a very extenfive fyftem of maftication;

* BLUMENBACH, in the Philof. Tranfact. 1794, Part II. p. 182, makes fome interefting remarks on the form of the teeth of different nations, and on thofe of the ancient Egyptians, according to their mummies, which ftill remain. OLEARIUS, *iter Perfic. et Mofcov.* has alfo made fome obfervations on the teeth of Northern nations.

maſtication; which, independent of the climate they inhabit, proves, that they are deſigned by Nature to ſubſiſt chiefly on vegetables. Hence, the ſnouts of herbaceous quadrupeds are longer than thoſe of the carnivorous: in ſhort, the latter are more unmanageable, and become more vigorous, according to the quantity of fleſh they feed upon. The dog would ſoon become as ferocious as the wolf, if he lived in a ſimilar manner. The people of the North have always been more courageous, cruel, and ungovernable, than the peaceable inhabitants of the equatorial regions, whom they have often ſubdued.

The food derived from animals that have perfect organs of generation, however diſagreeable it may be to the taſte, eſpecially when the blood, under the influence of deſire, boils in their heated veins, is a violent ſtimulant, and increaſes the muſcular action and ſuſceptibility, conſiderably more than the ſoft and almoſt inſipid fleſh of caſtrated animals.

The fleſh of reptiles, *amphibia*, Linn. preſents as great a difference of food as thoſe of the preceding claſſes. Their bodies contain leſs albumen, leſs jelly, and are generally drier; their blood is likewiſe not ſo rich in craſſamentum,* and their irritability is more adherent; a circumſtance which ſeems to depend on their organization, and particularly on reſpiration, and the circulation of the blood.

The fat of ſea turtles is green, and their eggs contain ſo ſmall a quantity of albumen, that the white does not coagulate by heat. The fleſh and eggs of the crocodile, ſmell like muſk, and this odour is alſo found in ſeveral ſerpents, particularly in their excrements. The ſpawn of frogs is perfectly gelatinous. Beſides the ſingular property of ſeveral reptiles, that change their colour by different affections, many ſtriking ſingularities are diſcoverable in the uſe of their fleſh. That of the tortoiſe turtle, *Teſtudo Caretta*, L. and eſpecially that of the *scinc*, have been extolled as aphrodiſiacs. If theſe animals poſſeſs that property, it is probably derived from their feeding on inſects, which, it is well known, powerfully ſtimulate the kidneys and bladder. Trnka praiſes the fleſh of ſeveral reptiles, as efficacious in hectic fever.

It ſeems to be demonſtrated by a number of obſervations, that the internal uſe of theſe animals, carries to the ſurface of the human body, the different virus with which it is infected, ſuch as the leproſy, and other cutaneous maladies, but particularly the venereal diſeaſe. It has alſo been aſſerted, that reptiles were

* The blood of theſe animals coagulates only by means of fire.—Oexmelin, Hiſt. Avent. Tom. I. p. 99. relates the ſame of the turtle.

were hurtful in lues, becaufe they occafioned a relapfe before it had been perfectly cured.

It is not my intention, at prefent, to treat on the poifons of animals, left they fhould be confounded with alimentary fubftances.

Moft Mahometans, particularly thofe of the fect of Ali, confider reptiles as unclean: only the pooreft people ufe them as food; and in the province of Thebes, the lower clafs of the inhabitants fubfift chiefly on lizards.

Mythology informs us, that Nereus was the firft who inftructed mankind to feed on fifh. Thefe animals have red blood, and vertebrae; they are innumerable, and confequently afford abundant aliment. They are eafily caught, are agreeable food, require but little trouble in preparing, and abound in oleaginous fubftances. Such are the principal advantages which fifh afford to the people of Kamtfchatka, the Samoiedes, the Dutch, and many other fifhing and maritime nations. Numbers of perfons eat them raw, fmoked and dried, or even frozen and putrid! Others drink large draughts of their naufeous oil with avidity. Could we, in the prefent age, relifh that putrified feafoning, the *garum* of the ancients?

> Expirantis adhuc Scombri de fanguine primo
> Accipe foecofum munera chara garum.
>
> Martial.

Several of the weftern nations fubftitute for *garum* the caviare, or the *botargo* of the Chriftian Greeks. In the prefent age, however, we do not gormandize thefe fifh to fuch excefs as the luxurious Romans did. Our modern Pollios have not yet caufed their flaves to be devoured by lampreys, and we do not go into mourning for the death of the latter. It has been afferted, that the eggs of fome fifh, as thofe of the barbel, are draftic; this error has been refuted by Bloch. Ichthyologifts no longer believe, that herrings fally forth in battle array, from the bottom of the Northern ocean: the fexual impulfe alone brings them to our fhores. It is the fame with falmon, the red flefh of which becomes white, when the act of reproduction is accomplifhed. Aquatic birds do not digeft the eggs of fifh, nor do they even deprive them of the power of developing themfelves. Nature feems to have appointed thofe birds to be the diffeminators of fifh.

The foft kinds of fifh, fuch as the malacopterygians, and chondropterygians of Artedi, afford unwholefome and often putrid aliment, particularly in thofe torrid regions where there is continual fun-fhine: hence the Hebrew legiflator forbade the ufe of fifh without fcales, as are moft of thofe above-mentioned.

They

They certainly furnish a large quantity of light jelly, especially in their skin,* and which communicates to their whole body a slimy and vapid taste.

[ To be continued in our next Number. ]

* OLAUS BORRICHIUS has discovered in the cod more carbonat of ammonia (concrete volatile alkali,) than in any terrestrial animal. Hence he concludes, that fish possess most hydrogen, and the other animals most azote.

